# A bioinformatics analysis: ZFHX4 is associated with metastasis and poor survival in ovarian cancer

**DOI:** 10.1186/s13048-022-01024-x

**Published:** 2022-08-01

**Authors:** Shuai Zong, Ping-ping Xu, Yin-hai Xu, Yi Guo

**Affiliations:** 1 Department of Laboratory Medicine, Affiliated Hospital of Xuzhou Medical University, No. 99 West Huaihai Road, Xuzhou, Jiangsu 221002 People’s Republic of China; 2 Department of Laboratory Medicine, Xuzhou Central Hospital, Jiangsu, 221006 China

**Keywords:** ZFHX4, Ovarian cancer, Metastasis, Epithelial-mesenchymal transition, Extracellular matrix

## Abstract

**Background:**

Metastasis was the major cause of the high mortality in ovarian cancer. Although some mechanisms of metastasis in ovarian cancer were proposed, few have been targeted in the clinical practice. In the study, we aimed to identify novel genes contributing to metastasis and poor clinical outcome in ovarian cancer from bioinformatics databases.

**Methods:**

Studies collecting matched primary tumors and metastases from ovarian cancer patients were searched in the Gene Expression Omnibus (GEO) database. Differentially expressed genes (DEGs) were screened by software *R* language. Gene Ontology (GO) enrichment and Kyoto Encyclopedia of Genes and Genomes (KEGG) pathway analysis for the DEGs were implemented by Metascape. Venn diagram was plotted to present overlapping DEGs. The associations between the overlapping DEGs and prognosis were tested by Cox proportional hazard regression model using a cohort of ovarian cancer patients from the TCGA database. Genes affecting patients’ outcomes significantly were served as hub genes. The mechanisms of the hub genes in promoting ovarian cancer metastasis were then predicted by *R* software.

**Results:**

Two gene expression profiles (GSE30587 and GSE73168) met the inclusion criteria and were finally analyzed. A total of 259 genes were significantly differentially expressed in GSE30587, whereas 712 genes were in GSE73168. In GSE30587, DEGs were mainly involved in extracellular matrix (ECM) organization; For GSE73168, most of DEGs showed ion trans-membrane transport activity. There were 9 overlapping genes between the two datasets (RUNX2, FABP4, CLDN20, SVEP1, FAM169A, PGM5, ZFHX4, DCN and TAS2R50). ZFHX4 was proved to be an independent adverse prognostic factor for ovarian cancer patients (HR = 1.44, 95%CI: 1.13–1.83, *p* = 0.003). Mechanistically, ZFHX4 was positively significantly correlated with epithelial-mesenchymal transition (EMT) markers (*r* = 0.54, *p* = 2.59 × 10^−29^) and ECM-related genes (*r* = 0.52, *p* = 2.86 × 10^−27^).

**Conclusions:**

ZFHX4 might promote metastasis in ovarian cancer by regulating EMT and reprogramming ECM. For clinical applications, ZFHX4 was expected to be a prognostic biomarker for ovarian cancer metastasis.

## Introduction

Ovarian cancer is the leading cause of gynecological cancer death in the developed countries and the second mortal gynecologic malignancy in China [1, 2]. Extensive metastasis due to late diagnosis and refractory relapse, which always presented as widespread intraperitoneal dissemination, is chiefly responsible for this discouraging statistic [3, 4]. Therefore, the exploration of the mechanism of metastasis has been put under the spotlight in the investigations for ovarian cancer. Rodriguez et al. found that canonical TGF-β signaling could significantly increase metastatic potential of ovarian cancer cells as early as 2001 [5]; Extracellular matrix (ECM) remodeling, a recent research hotspot, was perceived as contributing to ovarian cancer metastasis as well [6]. Other mechanisms associated with ovarian cancer metastasis were also widely proposed [7], but few have been targeted or fully-validated in the clinical practice. For instance, the duel nature of TGF-β in tumor suppressing and tumor promoting limited its role in judging the prognosis of ovarian cancer patients [8]; Matrix metalloproteinase inhibitors (MMPIs) that were designed to control ECM degradation did not improve patient survival due to lack of specificity and severe side effects [9].

Currently, with the rapid development of molecular expression profiling technologies, such as gene microarray, next generation sequencing and protein chips, and bioinformatics tools, several research groups have successfully predicted clinical outcomes and guided treatment decisions for cancer patients using bioinformatics data. Mannaprint® assays identified poor outcome patients early in the course of breast cancer by results from trancriptome profiling [10]. Using machine-learning techniques on the SAMIT trials, Sundar et al. developed a gene signature, which could represent as predictive biomarker for paclitaxel benefit in gastric cancer [11]. Liu et al. screened out five genes as a panel of biomarkers from sequencing data, and demonstrated that the panel could stratified EGFR-mutant NSCLC patients and provided precise guidance for them in the ADJUVANT trial [12]. These researches provided directions for us to search for novel genes and pathways involved in ovarian cancer metastasis.

Therefore, in the study, we aimed to find out new hub genes which were fit for drug targets and biomarkers in ovarian cancer metastasis, and probe into underlying mechanisms by mining bioinformatics databases. For the purposes, we searched the studies including samples of primary tumors and metastatic lesion in the Gene Expression Omnibus (GEO) database, and differentially expressed genes (DEGs) between groups were, then, screened by programming software *R* language. This was followed by Gene Ontology (GO) functional analysis and Kyoto Encyclopedia of Genes and Genomes (KEGG) pathways enrichment of DEGs. Next, the hub genes were identified by using Venn diagram and Cox regression analysis, and the functions of the hub genes in ovarian cancer metastasis were explored using samples from TCGA database with *R* software.

## Materials

### Strategies of search

We systematically searched for the relevant studies in the GEO database (https://www.ncbi.nlm.nih.gov/gds/). The search terms were shown as follow: [("Ovarian Neoplasms") OR ("Ovarian Neoplasm") OR ("Ovary Neoplasms") OR ("Ovary Neoplasm") OR ("Ovary Cancer") OR ("Ovary Cancers") OR ("Ovarian Cancer") OR ("Ovarian Cancers") OR ("Cancer of Ovary") OR ("Cancer of the Ovary")] AND [("Metastasis") OR ("Metastases") OR ("Metastatic")]. The inclusion criteria for eligible studies were that (1) the enrolled population was treatment-naive patients with ovarian cancer; (2) matched primary tumors and metastatic lesions from ovarian cancer patients were collected and analyzed; (3) platforms used for sequencing or gene chip were introduced. The exclusion criteria were that (1) ovarian cancer patients in the study were secondary to / combined with other malignancies; (2) gene probe ID in certain platforms could not be transformed into gene names; (3) the data in the downloaded files were incomplete or invalid.

### Selection of DEGs

The raw data were downloaded as Series Matrix Files. The data values in the microarray were log_2_-transformed, and normalized using function *normalize.quantiles* in *R* programming tools (version 4.2.0). Probes were converted to gene symbols according to the annotation information of the platform. Then, “limma” package in the *R* language (version 4.2.0) was used to screen DEGs. The thresholds for DEGs identification were set as *p* value < 0.05 and absolute fold change ≥ 1.5. Volcano plots and heat maps were generated using TBtools (version 1.049), which is an integrative toolkit developed for interactive analyses of big biological data [13].

### Analysis of functions and pathways

To predict potential biological functions of DEGs and pathways they might be involved in, we using Metascape (https://metascape.org/) to perform annotation analysis. Metascape was a web-based tool, and has been designed for experimentalists to apply powerful computational analysis pipelines to analyze and interpret large-scale datasets [14]. Using Metascape, we implemented the GO enrichment for the lists of DEGs. Deregulated pathways were also ranked on the basis of − log_10_ (*p* value) from KEGG analysis by Metascape.

### Identification of hub genes

Firstly, Venn diagram was plotted online to present common DEGs (http://jvenn.toulouse.inra.fr/app/example.html), and the overlapping DEGs were regarded as candidate genes for further analysis. Next, the associations between candidate genes and prognosis were tested by Cox proportional hazard regression model on the cohort of ovarian cancer patients from the TCGA database. Univariate and multivariate cox regression analysis were performed using “forestplot” package from *R* software (version 4.2.0). A nomogram was developed to predict the 1, 3, and 5-year overall survival using “rms” package in *R* programming language (version 4.2.0). The nomogram was validated by calibration curves using the same *R* package. We served genes affecting patients’ outcome significantly (*p* < 0.05) as hub genes. Finally, Kaplan–Meier analysis was used to verify the effect of hub genes on survival using Kaplan–Meier plotter (http://kmplot.com/analysis/).

### Exploration of mechanisms of hub genes in ovarian cancer metastasis

The functions of the hub genes and the signaling pathways they might be involved in were explored using samples from TCGA database. “GSVA” package in *R* software (version 4.2.0) was used to analyze the correlation between hub genes and pathway scores by Spearman correlation [15], and *p* values less than 0.05 were considered statistically significant. The associations of hub genes and other gene sets were also performed using the same *R* package.

## Results

### Data source

One hundred and ten studies were retrieved from the GEO database. After screening titles and abstracts, 101 studies were excluded. Among them, 82 studies did not select matched primary tumor and metastasis, 14 studies included patients combined with other malignant tumors, 3 studies uploaded the incomplete data, and 2 studies contained patients received neo-adjuvant chemotherapy before operations. In the remaining 9 studies, 5 did not provide enough platform annotation information for us to convert EntrezID into gene names, so 4 records, which contains GSE30587, GSE73064, GSE73091 and GSE73168, respectively, met the inclusion criteria. Because GSE73064 and GSE73091 were subgroups of GSE73168, 2 gene expression profiles (GSE30587 and GSE73168) were finally analyzed.

GSE30587 consisted of 9 pairs of matched primary and metastatic tumors from ovarian cancer patients, and metastatic lesions were located in the omenta. GSE73168 contained 8 pairs of matched lesions, and metastases were isolated from peritonea. The chip-based platform GPL6244 (HuGene-1_0-st; Affymetrix Human Gene 1.0 ST Array) was applied for the GSE30587, and the data from GSE73168 was generated from GPL570 platform (HG-U133_Plus_2; Affymetrix Human Genome U133 plus 2.0 Array).

### Gene Expression Changes in GSE30587 and GSE73168

**Fig. 1:**
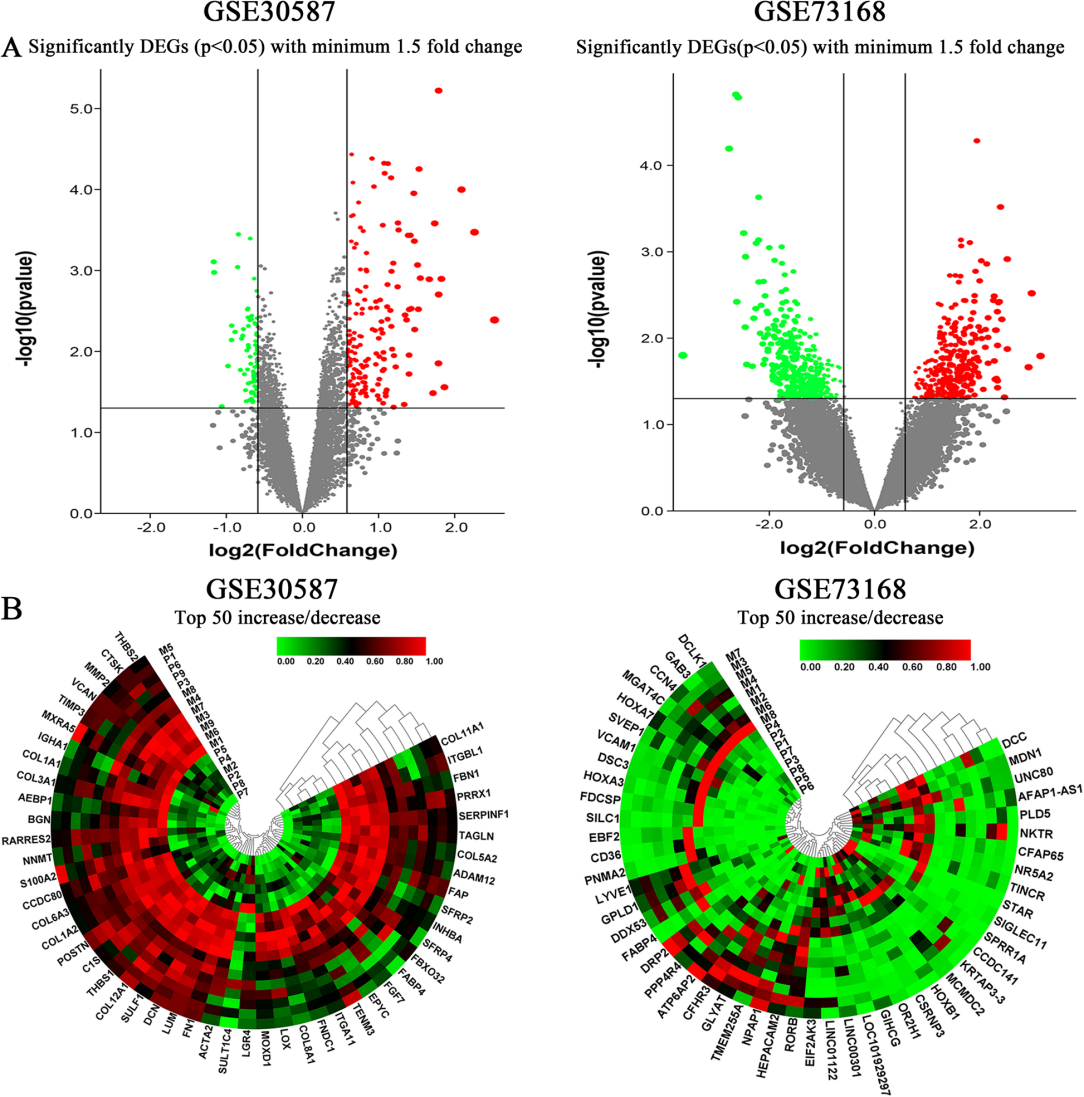
Gene expression changes in ovarian cancer metastasis. **A** Volcano plot representing DEGs in GSE30587 and GSE73168. **B** Heat map representing top 50 increase and decrease DEGs in GSE3058 and GSE73168. Note: DEGs: differently expressed genes

A total of 259 genes were significantly differentially expressed in GSE30587 with a significant level of *p* value < 0.05 and absolute fold change≧1.5 (Fig. 1A Left), whereas 712 genes were differentially expressed in GSE73168 (Fig. 1A Right). The green dots represented down-regulated genes, while the up-regulated genes were red-color coded. The top 50 up-regulated and down-regulated genes were then determined and showed as heat maps (Fig. 1B).

**Fig. 2:**
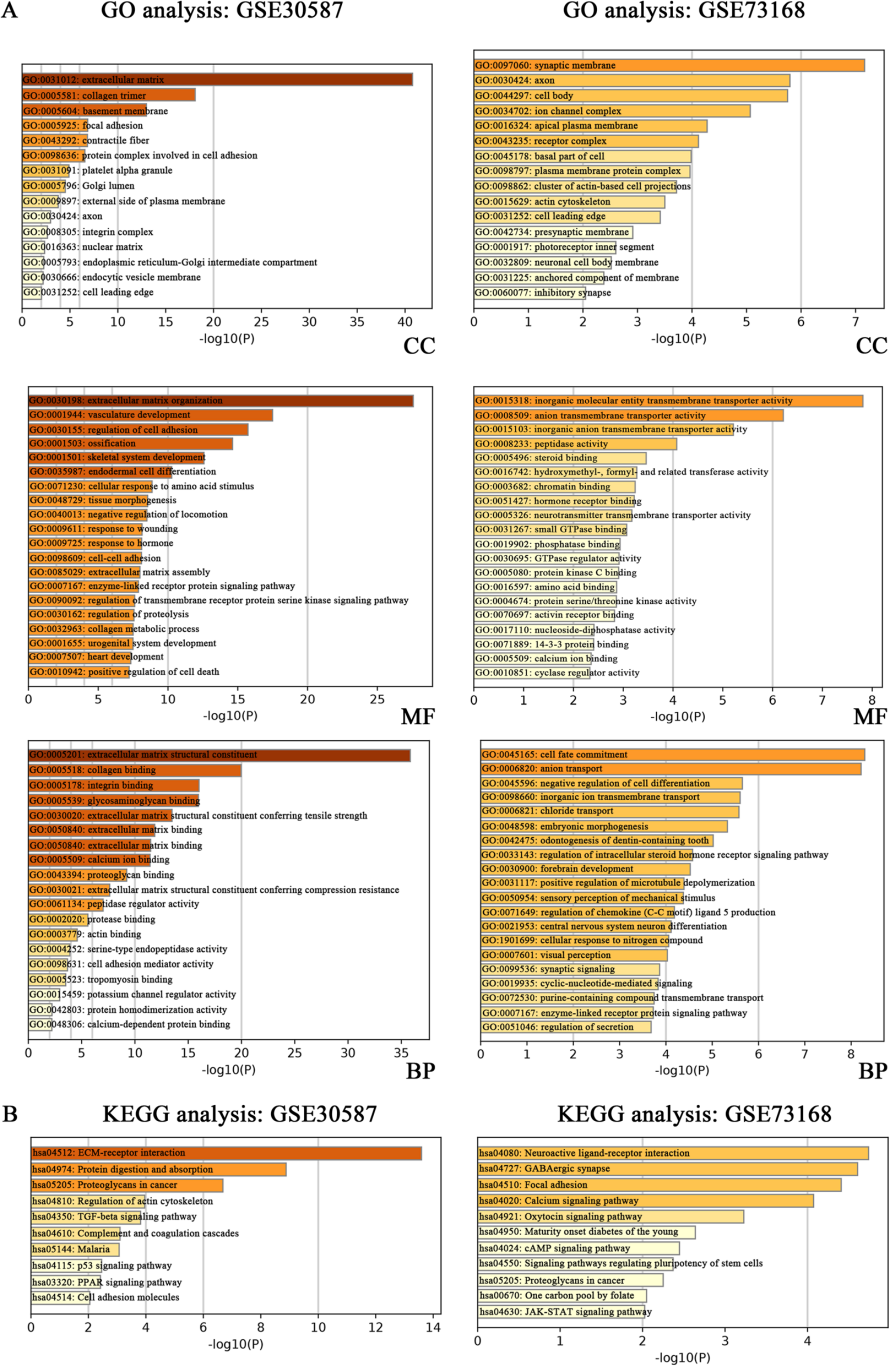
Function analysis of deregulated genes. **A** GO enrichment analysis of DEGs in GSE30587 and GSE73168. **B** KEGG analysis of DEGs in GSE30587 and GSE73168. Note: GO: Gene Ontology; DEGs: differently expressed genes; CC: cellular component; MF: molecular function; BP: biological process; KEGG: Kyoto Encyclopedia of Genes and Genomes

GO analysis was done to annotate the biological functions DEGs occurring during metastasis. In GSE30587, DEGs were mainly located in the extracellular matrix (ECM) of cellular components (CC), exhibited the molecular functions (MF) of ECM structural constituent, and were involved in the biological processes (BP), such as extracellular matrix organization, collagen binding, and integrin binding (Fig. 2A Left). For GSE73168, DEGs were primarily enriched in plasma membranes, showed functions of anion trans-membrane transporter activity, and participated in the processes of inorganic ion trans-membrane transport (Fig. 2A Right). Remarkably, terms related with development, for instance, vasculature development, heart development, cell fate commitment, skeletal system development, embryonic organ development, regulation of wound healing and tissue remodeling, were common in both of the two data sets (Fig. 2A).

KEGG analysis was done to find out deregulated pathways involved in metastasis. Complement and coagulation cascades and ECM-receptor interaction were the most significant pathways in the GSE30587 (Fig. 2B Left), while the most significantly correlated pathways in GSE73168 were Neuroactive ligand-receptor interaction (Fig. 2B Right). GO enrichment and KEGG pathway analysis offered guides for exploring the functions of key genes screened out for subsequent experiments.

### Overlapping Genes in GSE30587 and GSE73168

**Fig. 3:**
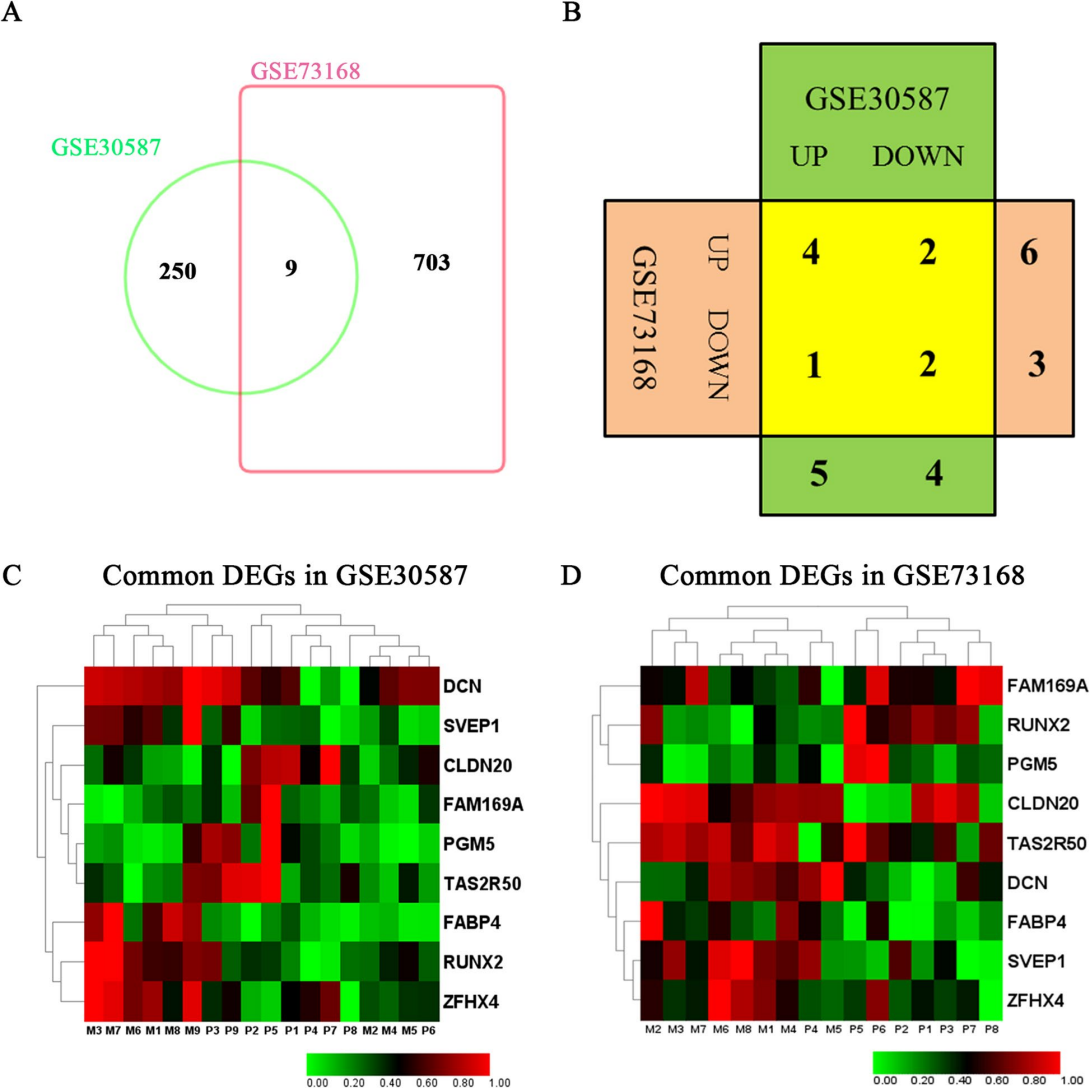
Common Genes between GSE30587 and GSE73168. **A** Venn diagram representing the overlapping genes between GSE30587 and GSE73168. **B** Table representing common upregulated and downregulated genes between GSE30587 and GSE73168. **C** The expression of the common genes in GSE30587. **D** The expression of the common genes in GSE73168

It is expected that the expressions of various genes altered as the tumors progressed from primary sites to metastasis loci. As mentioned above, GSE30587 showed that the process of metastasis resulted in deregulation of 259 mRNAs, while the dissemination formation was accompanied by changes of 712 mRNAs in GSE73168. In the next step, we characterized the overlap in the two datasets. Of the 259 and 712 DEGs in GSE30587 and GSE73168, respectively, 9 overlapping genes were identified (Fig. 3A). Four genes were commonly up-regulated, two genes were down-regulated in both of the datasets, whereas other three genes showed different directions of change in the two groups two (Fig. 3B). The co-upregulated DEGs included FABP4, SVEP1, ZFHX4 and DCN, FAM169A and PGM5 were co-downregulated, CLDN20 and TAS2R50 were down-regulated in GSE30587 but up-regulated GSE73168, and RUNX2 was up-regulated in GSE30587 but down-regulated in GSE73168 (Fig. 3C and D). The nine overlapping genes were considered as candidate genes for further analysis.

### Univariate and multivariate analysis of ovarian cancer patients in the TCGA database

To validate the effects of candidate genes on the outcome of ovarian cancer, we conducted univariate and multivariate Cox proportional hazards analysis on 374 patients from the TCGA database. The characteristics of the patients in the cohort were included in Table 1. The median follow-up was 86.0 months. At the end of follow-up, a total of 230 (61.5%) deaths occurred. The median age at diagnosis was 59 years (range: 30–87). Patients with stage III/IV ovarian cancer accounted for 94.1%.

**Table 1:** Clinical information of ovarian cancer patients in the TCGA database

	**Characteristics**	**N (%)**
**Status**	Alive	144 (38.50)
	Dead	230 (61.50)
**Age**	Median (Min, Max)	59 (30, 87)
**Gender**	Female	374 (100.00)
**Race**	American Indian	8 (2.14)
	Asian	11 (2.94)
	Black	25 (6.68)
	Islander	6 (1.61)
	White	324 (86.63)
**TNM stage**	IC	2 (0.53)
	IIA	3 (0.81)
	IIB	5 (1.34)
	IIC	15 (4.01)
	IIIA	7 (1.87)
	IIIB	14 (3.74)
	IIIC	271 (72.46)
	IV	57 (15.24)
**Grade**	G1	6 (1.60)
	G2	42 (11.24)
	G3	320 (85.56)
	G4	6 (1.60)

Age was presented using median and range

**Fig. 4:**
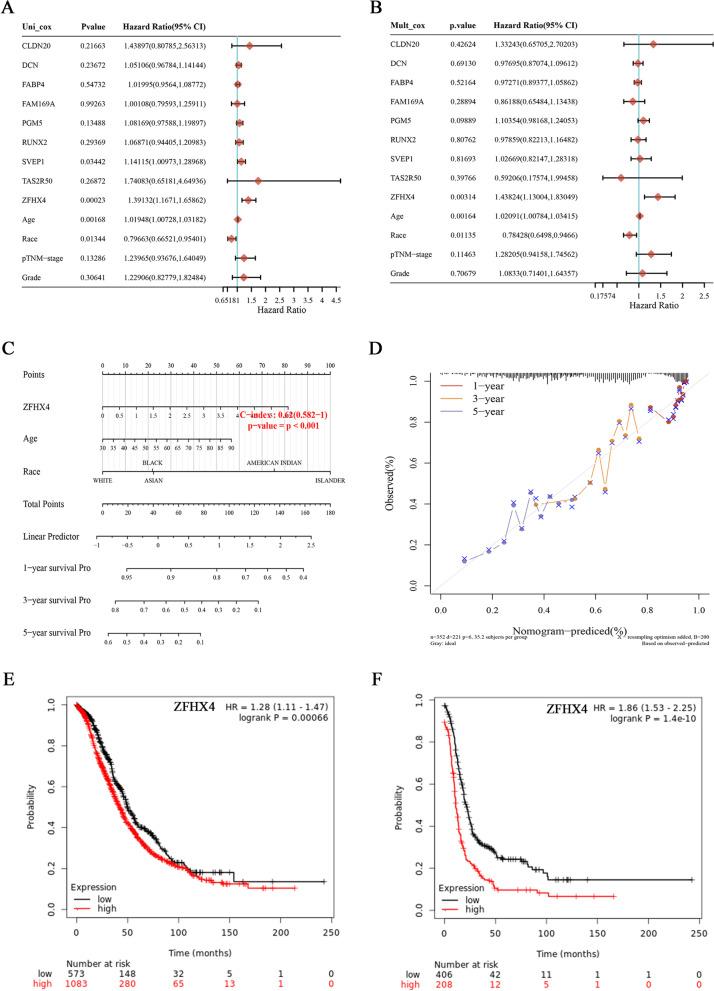
Hub genes selection using univariate and multivariate analysis. **A** Univariate analysis of prognostic factors of OS in ovarian cancer patients. **B** Multivariate analysis of prognostic factors of OS in ovarian cancer patients. **C** The nomogram for predicting probabilities of patients of 1, 3, and 5-year OS. **D** Evaluation of ZFHX4 prediction model by calibration plots. **E** Kaplan–Meier plot for OS analysis for ZFHX4. **F** Kaplan–Meier plot for PFS analysis for ZFHX4. Notes: OS: overall survival; PFS: progress-free survival; Islander: Pacific Islander

Univariable analysis indicated that ZFHX4 was the prognostic predictors among the candidate genes (Fig. 4A). In multivariable analysis, ZFHX4 was still proved to be an independent adverse prognostic factor for ovarian cancer patients (H*R* = 1.44, 95%CI: 1.13–1.83, *p* = 0.003) (Fig. 4B). A nomogram was plotted to predict 1-, 3-, and 5-year OS of ovarian cancer patients. As shown in the Fig. 4C, different values of ZFHX4 corresponded to various points. Total points could be calculated according to the patient’s ZFHX4, age and racial. Therefore, we could predict ovarian cancer patients’ OS by corresponding total points to survival probability. The calibration curve certified that nomogram-predicted curves were close to the observed curves, suggesting satisfactory agreement between the predicted results and the actual values (Fig. 4D).

We also checked the effect of ZFHX4 on ovarian cancer patient’s prognosis using Kaplan–Meier Plotter. Patients were spilt by median expression, and increased ZFHX4 caused significantly decreased OS (H*R* = 1.28, 95%CI: 1.11–1.47, *p* = 0.00066) and progress-free survival (PFS) (H*R* = 1.86, 95%CI: 1.53–2.25, *p* = 1.4 × 10^−10^) (Fig. 4E and F). ZFHX4, thus, was selected as the hub gene for the function exploration.

### Mechanisms of ZFHX4 in ovarian cancer metastasis

**Fig. 5:**
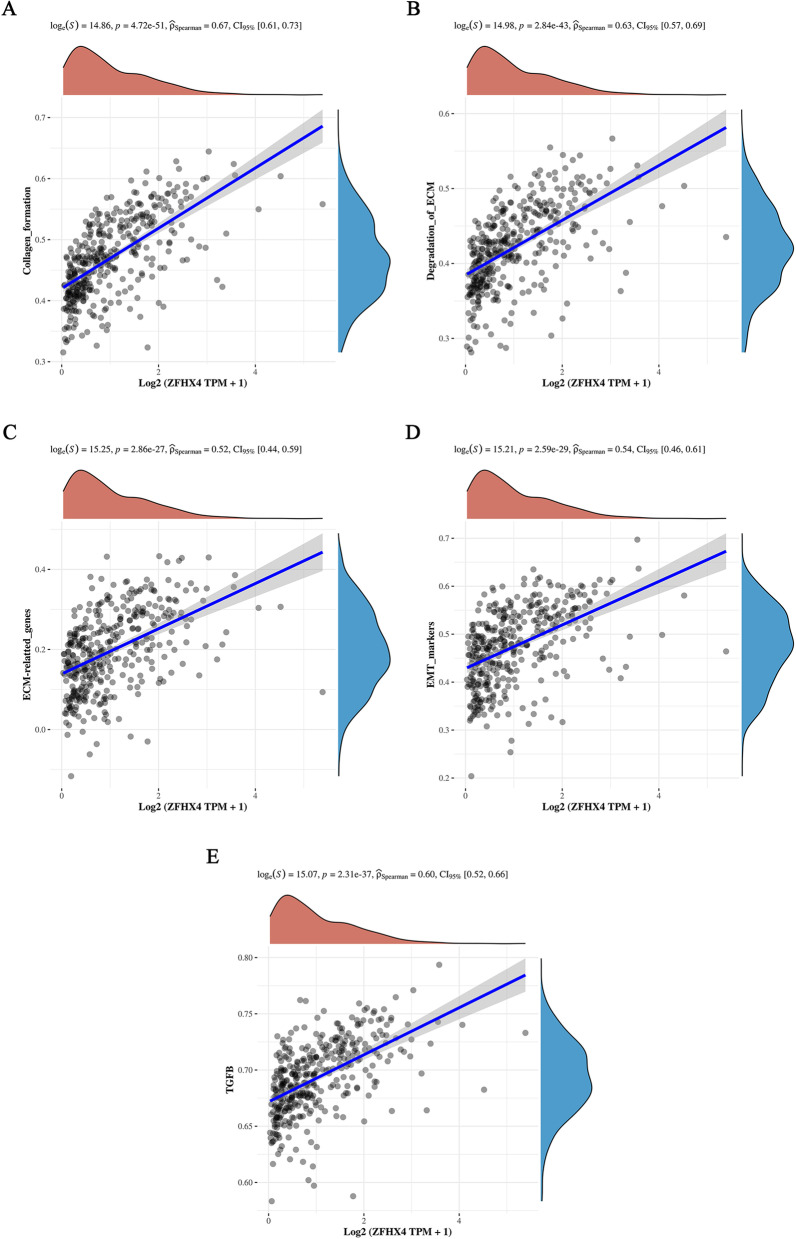
Functions and mechanisms of ZFHX4 in ovarian cancer metastasis. **A** The correlation between ZFHX4 and collagen-related genes. **B** The correlation between ZFHX4 and genes related to ECM degradation. **C** The correlation between ZFHX4 and ECM-related genes. **D** The correlation between ZFHX4 and EMT-related genes. **E** The correlation between ZFHX4 and TGF-β. Notes: ECM: extracellular matrix; EMT: epithelial-mesenchymal transition

To better understand the potential mechanisms of the hub gene in promoting metastasis in ovarian cancer, we explored the common pathways in which ZFHX4 might be involved in using ovarian cancer samples from TCGA database by R software GSVA package via ssGSEA algorithm. The most significantly positively correlated functions included collagen formation (*r* = 0.67, *p* = 4.72 × 10^−51^) and degradation of ECM (*r* = 0.63, *p* = 2.84 × 10^−43^) (Fig. 5A and B). ECM-related genes (*r* = 0.52, *p* = 2.86 × 10^−27^) and EMT markers (*r* = 0.54, *p* = 2.59 × 10^−29^) were the most significantly positively correlated gene sets (Fig. 5C and D). There was a significant positive correlation between TGF-β and the ZFHX4 (*r* = 0.60, *p* = 2.31 × 10^−37^) (Fig. 5E).

**Fig. 6:**
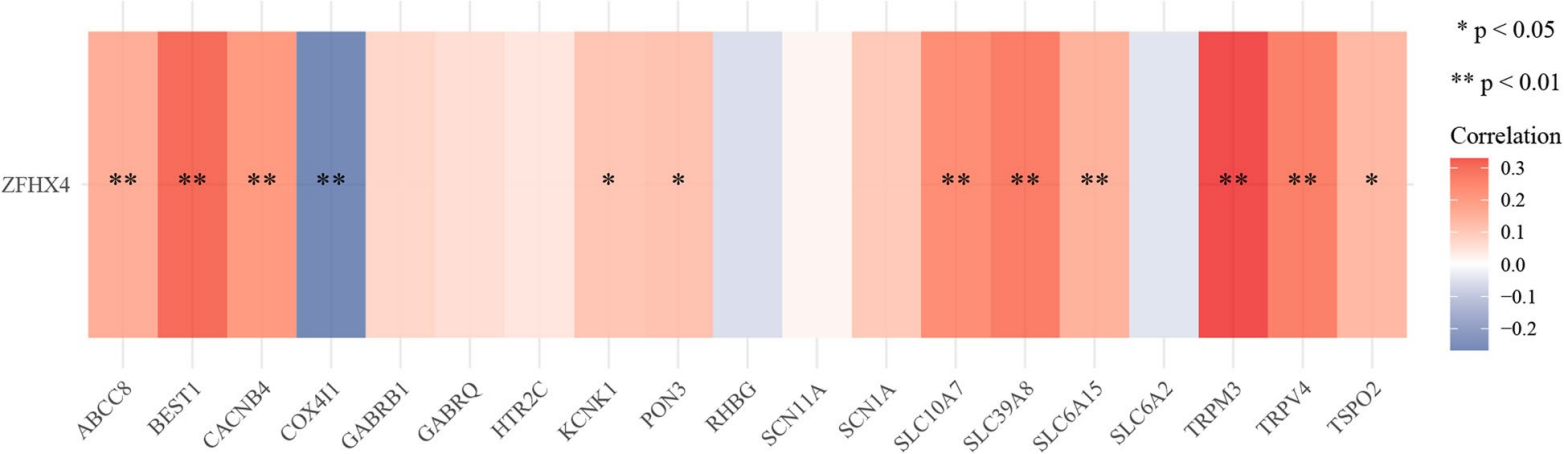
Correlations between ZFHX4 and genes encoding ion channel proteins. The correlations were visualized by color. The red color represented positive correlation, and the negative correlation was shown as blue color. The intensity of the color indicated the extent of correlation. Notes: The genes encoding ion channel proteins were obtained from Gene Ontology (GO) database (GO: 0,015,318)

As mentioned above, GO and KEGG analysis indicated that EMT and matrisome alteration were key features during ovarian cancer progression, which were in line with the predicted functions of ZFHX4. As enrichment analysis also implied that ion channels might play an important role in ovarian cancer metastasis, we further analyzed the relationships between ZFHX4 and ion trans-membrane transporters using gene sets from GO datasets (GO: 0,015,318). We found that TRPM3, BEST1, TRPV4, SLC39A8, SLC10A7, ABCC8, CACNB4, SLC6A15, TSPO2, KCNK1 and PON3 were significantly positively correlated with ZFHX4 (*p* < 0.05), while COX4I1 was significantly negatively correlated with ZFHX4 (*p* < 0.05) (Fig. 6). The results suggested that ZFHX4 might be regulated by the activation of ion trans-membrane channels.

**Fig. 7:**
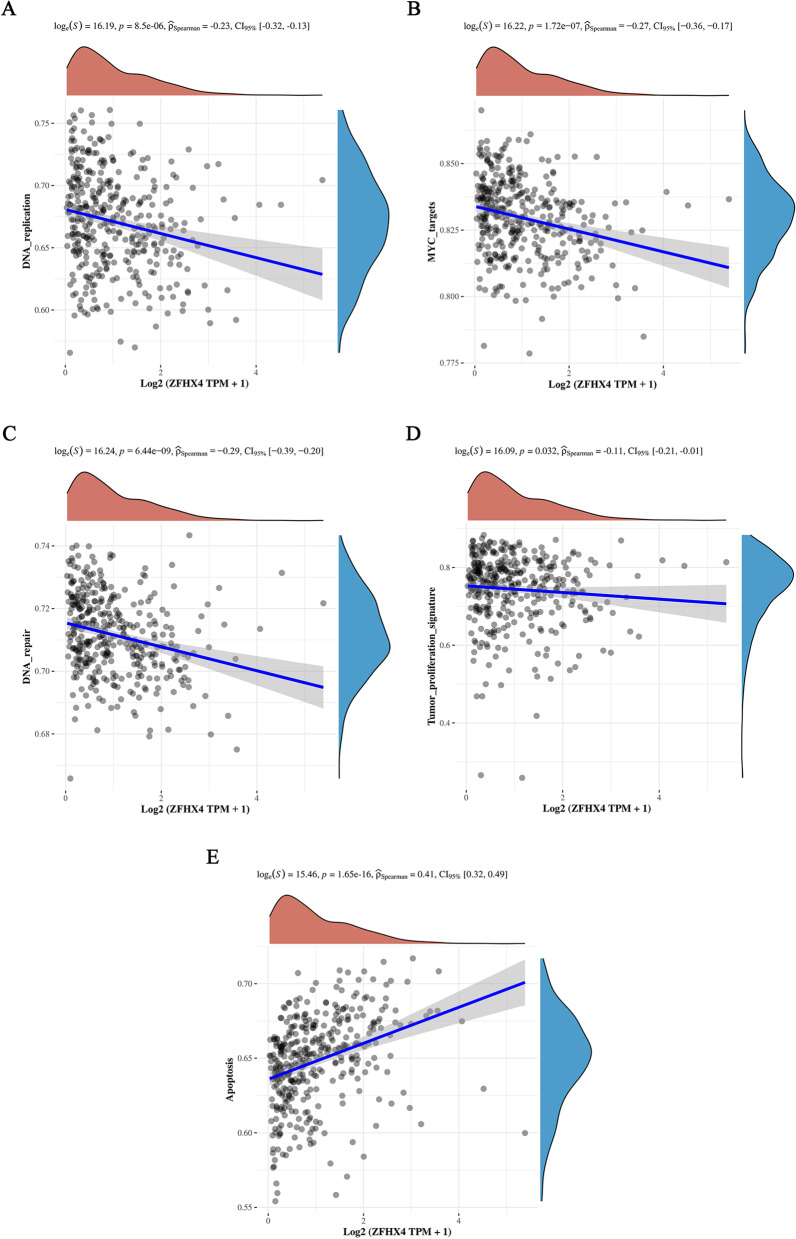
Functions of ZFHX4 in ovarian cancer proliferation. **A** The correlation between ZFHX4 and genes related to DNA replication. **B** The correlation between ZFHX4 and genes targeting MYC. **C** The correlation between ZFHX4 and genes related to DNA repair **D** The correlation between ZFHX4 and genes related to tumor proliferation. **E** The correlation between ZFHX4 and apoptosis-related genes

Notably, we also found that the expression of ZFHX4 was significantly negatively correlated with DNA replication (*r* =  − 0.23, *p* = 8.5 × 10^−06^), MYC targets (*r* =  − 0.27, *p* = 1.72 × 10^−07^), DNA repair (*r* =  − 0.29, *p* = 6.44 × 10^−09^) and tumor proliferation signature (*r* =  − 0.11, *p* = 0.032) (Fig. 7A, B, C and D), but significantly positively correlated with apoptosis (*r* = 0.41, *p* = 1.65 × 10^−16^) (Fig. 7E). These data suggested that ZFHX4 repressed the proliferative phenotype of ovarian cancer cells while it boosted their metastatic potentials.

## Discussion

In the present study, we did a bioinformatics analysis for primary tumors and metastatic lesions of ovarian cancer samples from GEO database to explore new genes and their mechanisms in ovarian cancer metastasis. While some information was available on the factors and pathways involved in the promoting invasiveness [16, 17], the progression of ovarian cancer was an intricate, multistage process along with numerous molecular changes, which further needed to be explored. Therefore, we systematically searched relevant studies in the GEO data library, and comprehensively analyzed the available datasets to find out common and unique signatures during the process of metastasis in ovarian cancer. Our results showed that the alterations of matrisome components and changes of gated channel activity were involved in this process. Moreover, we firstly identified ZFHX4 as the hub gene contributing to the ovarian cancer metastasis, and EMT regulation and ECM remodeling were considered as its potential mechanisms.

It has been well demonstrated that gene expression profiling of ECM components underwent deregulation during early colonization [18]. In turn, ECM remodeling could be conducive to cancer metastasis [19]. In the primary sites, basement membranes, which lined the basal surface of tumors and presented as dense collagen structures, were broken through proteolytic ECM degradation and non-proteolytic force by neoplastic cells and cancer-associated fibroblasts (CAFs) when tumor rapid growth led to diffusion-limited oxygen and nutrient supply [20–22]. Next, collagens in interstitial matrix were linearized by tumor-derived lysyl oxidase (LOX), which created migratory tracks used for the migration of the tumor cells [23, 24]. In the metastatic sites, however, CAFs deposited more collagens into matrix to support new lesions growth and help them evade immune surveillance [25, 26]. In accordance with the previous studies, we found that genes encoding alpha chains for fibrillar collagens, such as COL3A1, COL5A2, COL1A1 and COL1A2, were the top up-regulated DEGs in the metastatic samples compared to the primary ones. Besides collagens, altered glycoproteins, another kind of component in the ECM, might also contribute to the tumor metastasis. For instance, the expression of POSTN, which encoded a secreted extracellular matrix protein named Periostin, showed significant increase in the GSE30587. Yue.al reported that stromal Periostin could fuel the migration and invasion of ovarian cancer cells by binding to integrin αvβ3 and subsequently activating the PI3K/Akt pathway and inducing the EMT [27]. The regulation of TGFBI was disordered as well according to our results, and its encoding protein (transforming growth factor β induced protein, TGFBIp) was located in the extracellular matrix to bind to type I, II and IV collagens. As mentioned in the recent papers, macrophage-derived TGFBIp promoted ovarian cancer cell migration via inducing an immunosuppressive microenvironment [28, 29].

Ion channel disorder appeared to be responsible for the enhanced migration of cancer cells and ECM dysregulation [30, 31]. Aberrant activation of Ca^2+^-permeable channels on the endoplasmic reticulum membrane and plasma membrane led to impulse Ca^2+^ influx [32]. The oscillatory Ca^2+^ signaling facilitated invadopodial precursor assembly via Src activation and increased proteolytic activity of invadopodia by recruitment of ECM-degrading membrane type 1 matrix metalloproteinase (MT1-MMP) [33]. Then, Ca^2+^-activated K^+^ channels, such as SK3, enhanced the driving force for Ca^2+^ entry through hyperpolarization of resting membrane potential [34, 35]. Moreover, abnormal opens of sodium ion channels also contributed to invadopodia formation via F-actin polymerization [36]. Na^+^/H^+^ exchangers on the membrane caveola resulted in a per-imembrane acidification, which rendered secreted cathepsins and pH-dependent ECM-digesting proteases more active [37]. In our study, inorganic ion trans-membrane transports for calcium (TRPM3), sodium (SLC family) and potassium (KCNK1) were significantly up-regulated in the metastatic ovarian cancer samples compared to the primary ones, which were in accord with previous researches.

ZFHX4 is one of the five members of zinc finger homobox family with molecular weights of 397 kDa [38]. The protein comprises 22 typical C_2_H_2_ structures of zinc finger and 4 homeodomains, and its coding gene extends over 180 kb and is located on 8q13.3-q21.11[38]. ZFHX4 is predicted to be active in nucleus, and enable DNA-binding transcription factor activity. Fèvre-Montange et.al firstly reported that ZFHX4 was highly expressed in parapineal tumors, a rare but aggressive type of pineal region tumor, using oligonucleotide arrays in 2006 [39].Similarly, Wang et.al found that ZFHX4 amplifications were more common in the metastatic colorectal cancer patients than those without metastases [40]. In ovarian cancer, elevated expression of ZFHX4 was also demonstrated to be correlated with poor outcomes, which was mainly influenced by metastasis, in bioinformatics databases and real-world cohort samples [41, 42]. Our results were in accord with these studies, which indicated that ZFHX4 reduced the survival times of ovarian cancer patients through promoting metastasis.

Currently, researches for the functions of ZFHX4 largely focused on the therapy resistance and consequent recurrence. Chudnovsky et al. suggested that ZFHX4 interacted with the CHD4, an essential member of the NuRD (nucleosome remodeling and deacetylase) complex, to regulate the therapy-resistant tumor initiating cells in glioblastomas [43]. However, the detailed mechanism by which ZFHX4 regulated metastasis of ovarian cancer remained unknown. According to our bioinformatics analysis, we, for the first time, linked ZFHX4 with EMT-associated genes. In 2020, “the EMT International Association (TEMTIA)” issued a consensus statement on the famous academic journal “NATURE REVIEWS MOLECULAR CELL BIOLOGY” to provide guidelines and definitions for EMT [44]. In the statement, experts emphasized central roles of transcription factors belonging to the ZEB families (Zeb1 and Zeb2) in the execution of EMT in cancer metastasis [44]. Coincidentally, Zeb1 and Zeb2 were also two members of zinc finger homobox family, and they shared similar molecular structures with ZFHX4 [45], which rendered the function of ZFHX4 as an EMT inducer reasonable. Besides, ZFHX4 could be modulated by canonical pathways for EMT regulation, such as TGF-β signaling pathway [46, 47], which further supported the contributions of ZFHX4 to EMT. Simultaneously, we found the positive correlation between ZFHX4 and ECM-related genes. ZFHX4, on the one hand, was conducive to the collagen formation, but on the other hand, induced degradation of ECM. The results might be explained by the fact that individual cancer cell migrated faster in a mesenchymal phenotypes, while collective cell migration depended on the cell–matrix junctions [48]. Next, significant correlations were found between ZFHX4 and lots of ion channels. Eskandari et al. reported that Kv11.1 channel reprogrammed EMT in colon cancer via regulating zinc finger homobox proteins [49], suggesting that ZFHX4 might also act as a mediator converting the signals from ion transporter activation to EMT occurrence and ECM reprogramming in ovarian cancer. Although ZFHX4 took an active part in promoting cancer metastasis, it functioned as a tumor suppressor in tumor proliferation. This rather interesting finding might be explained by the opinion that expression changes of certain genes necessary for the initial tumorigenesis were not essential for subsequent metastasis, which was proposed by Mitra et al. [18]. In their study, they observed that most of shared deregulated genes metastasis versus primary tumors were altered in the different directions [18].

There were surely some limitations in the study. Firstly, DEGs were screened from primary tumors and metastatic lesions in GEO database, while the candidate genes were generated from ovarian cancer patients with various stages in TCGA database. Secondly, laboratory-based experiments have not been conducted to validate the mechanisms of ZFHX4 in metastasis in ovarian cancer. Thirdly, the interpretation of these data was without consideration of which cellular compartment expresses ZFHX4, since whole tumor samples were sequenced in GSE73168. In the future studies, clinical samples from ovarian cancer patients would be collected to detect the expressions of ZFHX4. Then, the relationships between ZFHX4 and EMT- and ECM-related proteins would be further confirmed by in vitro or (and) in vivo experiments.

## Conclusion

In summary, based on the bioinformatics analysis, we hypothesized that ZFHX4 might promote metastasis in ovarian cancer by regulating EMT and reprogramming matrisome. For clinical applications, ZFHX4 was expected to be a prognostic biomarker for ovarian cancer metastasis. On the one hand, ZFHX4 could be used to predict occurrence of metastasis in ovarian cancer patients, especially for those have completed therapy and were undergoing follow-up; On the other hand, the expression of ZFHX4 is prognostic for survival outcomes of ovarian cancer patients with metastasis.

## Data Availability

The datasets used and/or analyzed during the current study are available from the corresponding author on reasonable request.

## Abbreviations

GEO: Gene Expression Omnibus
DEGs: Differentially expressed genes
GO: Gene Ontology
KEGG: Kyoto Encyclopedia of Genes and Genomes
ECM: Extracellular matrix
EMT: Epithelial-mesenchymal transition
CC: Cellular components
MF: Molecular functions
BP: Biological processes
CAFs: Cancer-associated fibroblasts
LOX: Lysyl oxidase
MT1-MMP: Matrix 1-metalloproteinase
